# Improving Long-Term Outcomes for Patients with Extra-Abdominal Soft Tissue Sarcoma Regionalization to High-Volume Centers, Improved Compliance with Guidelines or Both?

**DOI:** 10.1155/2018/8141056

**Published:** 2018-04-03

**Authors:** Sanjay P. Bagaria, Yu-Hui Chang, Richard J. Gray, Jonathan B. Ashman, Steven Attia, Nabil Wasif

**Affiliations:** ^1^Department of Surgery, Section of Surgical Oncology, Mayo Clinic Florida, Jacksonville, FL, USA; ^2^Department of Biostatistics, Mayo Clinic Arizona, Phoenix, AZ, USA; ^3^Robert D. and Patricia E. Kern Center for the Science of Health Care Delivery, Surgical Outcomes Division, Mayo Clinic Arizona, Phoenix, AZ, USA; ^4^Department of Surgery, Section of Surgical Oncology, Mayo Clinic Arizona, Phoenix, AZ, USA; ^5^Department of Radiation Oncology, Mayo Clinic Arizona, Phoenix, AZ, USA; ^6^Division of Hematology and Oncology, Mayo Clinic Florida, Jacksonville, FL, USA

## Abstract

**Introduction:**

Optimization of outcomes of extra-abdominal STS is not clearly understood. We sought to determine whether hospital surgical volume and adherence to NCCN guidelines, or both, are associated with outcomes in the treatment of extra-abdominal soft tissue sarcoma (STS).

**Methods:**

The National Cancer Database (NCDB) was queried for patients undergoing surgery for extra-abdominal STS diagnosed from 2003 to 2007. Mean annual hospital volume for STS surgery was divided into volume terciles (1T ≤3, 2T 4–10, and 3T ≥11 cases/year). Adherence to NCCN guidelines was determined. Primary outcome was overall survival.

**Results:**

Our study population consisted of 13,684 patients with a median age of 56 years. 3T hospitals were more likely to adhere to NCCN guidelines for stage III patients (63% versus 47%; *p* ≤ 0.001) than 1T hospitals. On multivariable analysis, adherence to NCCN guidelines was associated with improved survival (HR = 0.79, CI 0.73–0.87; *p* < 0.001), but hospital volume was not (3T versus 1T: HR = 0.92, CI 0.82–1.02; *p*=0.12). Five-year overall survival was comparable for compliant groups at 1T, 2T, and 3T hospitals (72%, 72.4%, and 72.6%, resp.). 3T hospitals were not associated with a lower risk of 30-day mortality (OR 0.70, 95% CI 0.44–1.11) compared to 1T hospitals but did have a higher R0 resection rate (OR 1.43, 95% CI 1.32–1.54).

**Conclusions:**

Adherence to NCCN guidelines, irrespective of hospital volume, is associated with improved overall survival for patients with extra-abdominal STS. High-volume hospitals more often adhere to guidelines, but low-volume hospitals that follow national guidelines may achieve comparable outcomes.

## 1. Introduction

One of the challenges in managing complex cancers is identifying health care processes and structural measures that optimize outcomes. Hospital volume is a well-known measure that correlates with improved clinical outcomes for several cancer types [1]. Although this association has been most clearly demonstrated for postoperative mortality, improved long-term survival is also seen in high-volume hospitals [2]. Consequently, a strategy of regionalization that concentrates the management of complex cancers to high-volume centers has been endorsed by a number of investigators [3–6]. In practice, regionalization can be difficult to implement due to issues related to travel distance, patient choice, and disparities in access [7]. In contrast, the association of other health system variables with cancer outcomes, such as guideline concordant care and independent of volume status, has been less studied [8–10].

Soft tissue sarcoma (STS) is a group of over 50 rare mesenchymal malignancies that accounts for less than 1% of all new cancers. Optimal treatment of STS is based on an institution's experience and resources to provide a multidisciplinary effort to treat such a rare and complex malignancy. Although a volume-outcome relationship has been demonstrated for STS, optimizing cancer outcomes likely also depends on the appropriate use and expertise of a multimodality team that includes pathologists, radiation oncologists, medical oncologists, and surgeons [9, 11]. However, it is not clear if improved outcomes observed at high-volume centers are secondary to surgical volume, physician expertise, and/or increased compliance with guidelines. Answering this question is important to inform the debate on whether all sarcoma care in the United States should be regionalized to high-volume cancer centers, or more attention should be directed towards improving adherence to guidelines at all hospitals caring for these patients.

Consequently, the purpose of this study was to analyze the impact of hospital surgical volume and adherence to NCCN guidelines on outcomes for extra-abdominal STS patients. We hypothesize that increased adherence to NCCN guidelines is associated with higher overall survival (long-term outcomes) and that high hospital surgical volume is associated with improved 30-day mortality and R0 resection rates (short-term outcomes).

## 2. Methods

### 2.1. Data Source

Data from the National Cancer Database (NCDB) was used to conduct this study. The NCDB, a joint program of the Commission on Cancer (CoC) of the American College of Surgeons (ACoS) and the American Cancer Society (ACS), is a nationwide oncology outcomes database for more than 1,500 commission-accredited cancer programs in the United States and Puerto Rico. Approximately 70% of all newly diagnosed cases of cancer in the United States are captured at the institutional level and reported to the NCDB. Variables in the database cover demographics, socioeconomic status, tumor stage, treatment received, and hospital characteristics. NCDB data contain no protected health information; hence, this study was exempt from formal IRB review.

NCCN guidelines for extra-abdominal STS were accessed from the website as of February 8, 2017 (http://www.nccn.org). For this study, we utilized NCCN guidelines on the use of radiation therapy for these tumors with regard to margin status and tumor stage to divide patients into compliant and noncompliant groups (Table 1). These guidelines are the same as those in the study period. Though randomized control trials have not shown an improved overall survival with the use of radiation therapy in the setting of extra-abdominal STS, compliance to such guidelines may be a surrogate for interdisciplinary discussion and “best” practice medicine [12, 13].

### 2.2. Inclusion/Exclusion Criteria

Patients diagnosed with STS of the extremities, trunk, and head/neck from 2003 to 2007 were identified from the NCDB and constituted our study population (note that patients with retroperitoneal or intra-abdominal sarcoma were not included). This time period was chosen to ensure up to date coding in the NCDB for the variables of interest for this study and to provide at least 5 years of follow-up for survival analyses. Histologies included were liposarcoma, fibrosarcoma, leiomyosarcoma, malignant fibrous histiocytoma, myxofibrosarcoma, malignant peripheral nerve sheath tumor, and not otherwise specified (NOS). Patients with stage I, II, and III were included, and patients with stage IV were excluded. Only patients who underwent curative intent surgery were included in the study cohort. NCDB surgical codes distinguish between curative intent surgery and procedures such as open biopsies. Patients undergoing palliative surgery were excluded. In order to provide valid volume-outcome comparisons, only patients who had all treatment at the reporting hospital were included in the study cohort.

### 2.3. Outcome, Exposure, and Independent Variables

The exposure variables were hospital volume status and adherence to NCCN guidelines as outlined in Table 1. Primary outcome variable was overall survival, and secondary outcome variables were surgical margins and 30-day surgical mortality. Surgical margins were defined as microscopic negative (R0), microscopic positive (R1), or grossly positive (R2). Independent variables included age, sex, race, insurance status, education, modified Charlson score, primary tumor site, tumor size, grade, histology, radiation therapy, and chemotherapy.

### 2.4. Hospital Volume Calculations

We adapted the methods from Birkmeyer et al. to compute the hospital volume [14]. The number of cases performed at a hospital was computed and treated as a continuous variable. The mean volume of a hospital was the total volume divided by the number of years a hospital reported to the NCDB. The mean annual STS surgery volume for each hospital was rounded up to an integer value. Our initial approach was to identify cutoffs in mean annual STS volume that would divide the patient population into three equal terciles (1T, 2T, and 3T). This was done *a priori* and before any analysis of the data was performed. Using this technique, the cutoff identified for high volume (i.e., 3T) was ≥11 cases per year.

### 2.5. Statistical Analyses

Bivariate analyses were initially performed to identify demographic, tumor, and treatment differences between different terciles of volume using the chi-square test or analysis of variance. Logistic regression analyses were used to model the margin negative resection and 30-day mortality following surgery, and adjusted odds ratios (OR) and 95% confidence intervals (CIs) were reported. We excluded patients who underwent amputation from the margin status analysis. Overall survival was estimated by the Kaplan–Meier method, and the comparison in the survival curves between different surgical volumes and compliance to NCCN guidelines was assessed by the log rank test. Cox regression analyses were used to model overall survival, and adjusted hazard ratios (HRs) and 95% CIs were reported. For the multivariable logistic regressions and Cox regression, we included demographic and clinical characteristics that were considered as variables influencing 30-day mortality and margin status. A *p* value of <0.05 was set as our threshold for statistical significance. The analysis was performed using SAS 9.4 and R 3.1.3.

## 3. Results

### 3.1. Demographics

Our study population consisted of 13,684 patients who underwent surgical resection for extra-abdominal STS (Table 2). The median age was 56 years, and 53% were male. Median follow-up was 62.5 months. The most common primary site was an extremity (53%), and the three most common histologic subtypes were not otherwise specified (37%), malignant fibrous histiocytoma (19%), and liposarcoma (18%). Approximately 52% patients underwent care that was adherent to NCCN guidelines regarding the use of radiation therapy.

### 3.2. Bivariate Analyses

Following the division of the study cohort by volume terciles, some noticeable differences between groups emerged (Table 2). Overall, most patients were treated in designated academic/research programs (54%). However, a pronounced difference in this distribution was seen between 1T and 3T centers: 87% of patients treated at 3T centers received care at academic hospitals compared to only 16% of patientes treated at 1T centers (*N*=7373). 3T centers were also more likely to see tumors that were >10 cm in size and hence had a higher proportion of patients with stage III tumors than 1T facilities (31.4% versus 21.8%; *p* < 0.001). The use of chemotherapy and radiation therapy was also higher in 3T facilities. Statistically significant differences in socioeconomic characteristics were seen between the volume terciles. Finally, patients treated at 3T centers were more likely to receive care in accordance to NCCN guidelines in the use of radiation therapy than at 1T centers (53% versus 50%; *p*=0.03). Though this finding is likely not clinically meaningful, these differences were most pronounced for stage III patients between 3T and 1T centers (59% versus 49%; *p* < 0.001). There was no difference in guideline compliance between 1T, 2T, and 3T centers in the management of stage I patients (51%, 52%, and 49%; *p*=0.47), or stage II patients (46%, 49%, and 53%; *p*=0.09).

### 3.3. Volume and Compliance Associations with Overall Mortality

**Figure 1:**
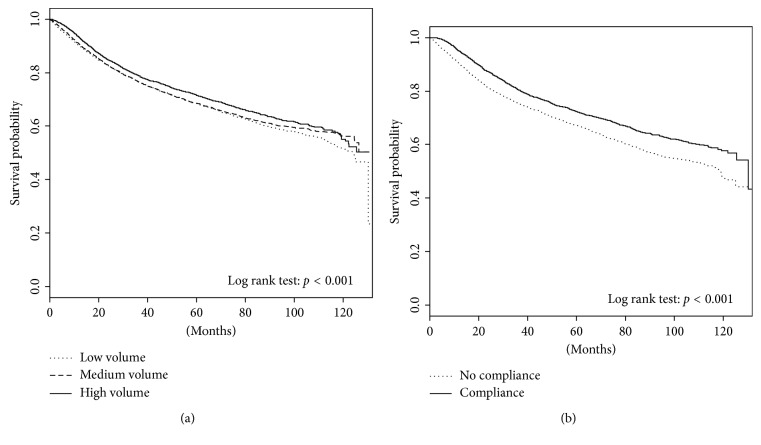
Kaplan–Meier plots of overall survival stratified by (a) hospital surgical volume and (b) adherence to NCCN guidelines.

A monotonic decrease in unadjusted overall mortality from 35.8% to 32.4% (*p*=0.003) was seen from 1T to 3T centers.  Figure 1 displays the Kaplan–Meier plots for overall survival stratified by hospital surgical volume (panel A) and adherence to NCCN guidelines (panel B). The 5-year overall survival rates for 1T, 2T, and 3T hospitals were 68.5%, 68.6%, and 71.5% (*p* < 0.001), respectively. The 5-year overall survival rates for NCCN guideline compliant and noncompliant patients were 72.4% and 67.2% (*p* < 0.001), respectively.

Table 3 demonstrates adjusted analyses for overall mortality. Adherence to NCCN guidelines was significantly associated with improved overall survival (HR = 0.79, 95% CI 0.73–0.87; *p* < 0.001). In contrast, hospital surgical volume was no longer associated with improved overall survival (HR 0.92, 95% CI 0.82–1.02; 3T versus 1T). As expected, larger tumor size, higher grade, and positive margins were all associated with a higher risk of overall mortality.

### 3.4. Adherence to NCCN Guidelines within Volume Strata

**Figure 2:**
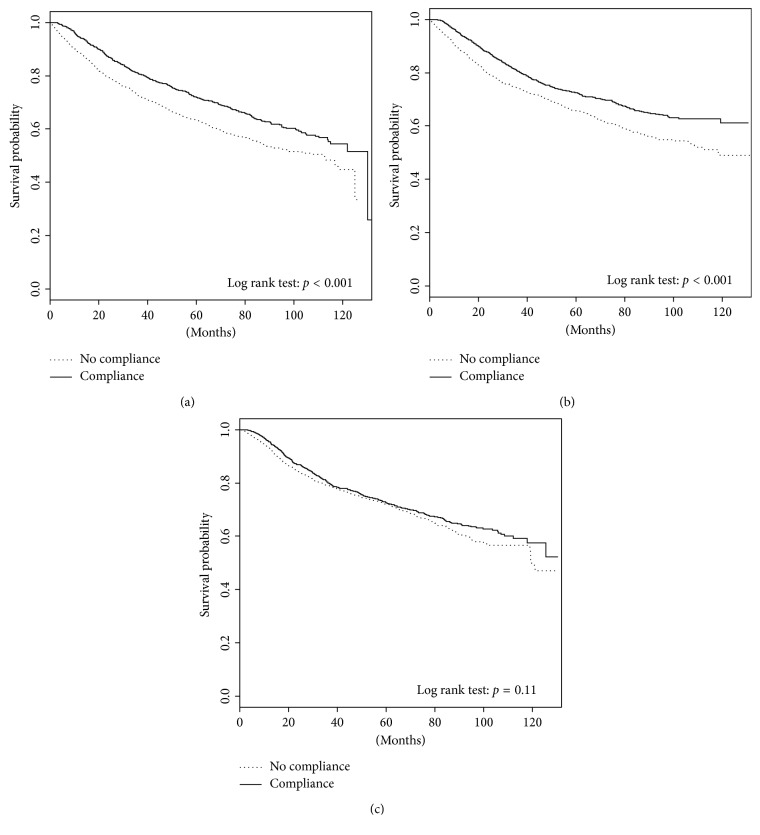
Kaplan–Meier plots displaying the impact of adherence to NCCN guidelines on overall survival stratified by hospital volume: (a) 1T hospitals, (b) 2T hospitals, and (c) 3T hospitals.

Figure 2 displays the relationship of adherence to NCCN guidelines within each strata of hospital volume with overall survival. The 5-year overall survival rate was significantly improved for patients treated at 1T hospitals who underwent NCCN compliant care compared noncompliant care (72.0% versus 63.4%; *p* ≤ 0.001). A similar association was seen for 2T hospitals, with improved 5-year overall survival for the compliant group compared to the noncompliant group (72.4% versus 65.8%; *p* < 0.001). There was no difference in 5-year overall survival for patients treated at 3T hospitals who received compliant care compared to those who received noncompliant care (72.6% versus 72.0%; *p*=0.11). It is important to note that 5-year overall survival for patients treated at 1T, 2T, and 3T hospitals was similar when treated in compliance with guidelines (72%, 72.4%, and 72.6%, resp.).

### 3.5. Factors Associated with 30-Day Mortality and Margin Negative Surgery

Short-term 30-day mortality showed a monotonic decrease from 1.2% for 1T centers to 0.4% for 3T centers (*p* < 0.001) (Table 2). However, on adjusted multivariable analysis (Table 4), the 30-day mortality rate at 3T centers was not significantly different to 1T centers (OR 0.46, 95% CI 0.15–1.35). Expected risk factors such as increasing age and higher modified Charlson comorbidity scores increased the risk of 30-day mortality following surgical resection.

The overall rate of margin negative resection was higher at 3T versus 1T centers (90% versus 83%, *p* < 0.001). After adjustment of variables, Table 5 demonstrates that patients who underwent surgical resection at either 2T or 3T hospitals were more likely to achieve margin negative surgery (defined as R0) when compared to patients in the 1st tercile (adjusted OR 1.77, 95% CI 1.51–2.08, 3T versus 1T, and OR 1.26, 95% CI 1.08–1.47, 2T versus 1T).

## 4. Discussion

Adherence to NCCN guidelines is a surrogate for “best practice” in a complex illness requiring a multidisciplinary subspecialty care. In this study, we analyzed the effect of adherence to NCCN guidelines in the use of radiation therapy and hospital surgical volume on outcomes for patients diagnosed with extra-abdominal STS. Consistent with previous studies, we show that high-volume centers were more likely to follow treatment guidelines than low-volume centers [15, 16]. This is not surprising since high-volume centers are more likely to have the necessary subspecialty care needed to treat extra-abdominal STS. We also demonstrate that, in a multivariable analysis, adherence to NCCN guidelines in the use of radiation therapy in the management of extra-abdominal STS significantly improves overall survival but hospital surgical volume does not. Patients treated under guidelines at low-volume centers were able to achieve comparable overall survival to those treated at high-volume centers. Finally, when we analyzed predictors of secondary outcomes, high-volume hospitals were associated with higher probability of margin negative resection but not lower 30-day surgical mortality.

In addition to multidisciplinary clinics and tumor boards, NCCN guidelines are a useful guide to provide treatment for patients with malignancies. One explanation for why adherence to NCCN guidelines had a greater impact on 1T hospitals as opposed to 3T hospitals is that 1T hospitals are less likely to have a multidisciplinary sarcoma team, and therefore NCCN guidelines are more valuable for 1T hospitals that do not have the expertise of 3T hospitals. In the 3T setting, where 87% of hospitals are an academic center, it is more likely that a multidisciplinary sarcoma team is directing care and following guidelines. A second explanation for why adherence to guidelines had a greater effect on overall survival is that they could actually reflect the direct impact of radiation therapy. Though phase III trials have not shown a survival benefit with radiation therapy, studies that use a larger sample size have shown a survival difference [12, 17–20]. Review of the NCDB database reported that the utilization of radiation therapy in the treatment of large, deep, high-grade STS is underutilized and that its use was associated with improved overall survival [19].

Thirty-day mortality at a 1T hospital was more than at a 3T hospital (1.2% versus 0.4%), yet the baseline overall rate remains low (0.9%). This is consistent with other published reports of low postoperative mortality following extra-abdominal STS surgery [21]. Consequently, the rare occurrence of a postoperative mortality in this setting is more likely related to patient comorbidities rather than to complications from the surgery itself, especially since adjusted analyses did not show a statistical difference between 3T and 1T centers. Unlike other high-risk cancer surgery, such as pancreatectomy or esophagectomy, the delta in 30-day mortality between high- and low-volume institutions for STS is not high enough to invoke this as a reason to centralize STS surgery.

One advantage of the NCDB over other registry data is the inclusion of margin status. It can be reported as either (a) margin negative or margin positive or (b) margin negative (R0), microscopic positive (R1), and macroscopic positive (R2). For the sake of simplicity, we used the R classification system and found that the margin negative rate (R0) of a 1T center was lower than that of a 3T center (83% versus 90%). This association persisted following adjustment for other variables related to margin status, such as tumor location and size, with 3T centers 77% more likely to achieve negative margins as 1T centers. Related to this, we also noted that 3T centers were more likely to treat more advanced cancers and perform amputation. It is possible that the higher margin positive rate noted for 1T centers may be secondary to their reluctance to perform an amputation. Whether such cases would have been better treated with amputation is not known. Also, it is possible that hospitals planned for positive margins for those cases where the tumor is in close proximity to vital neurovascular structures, a strategy that one would expect to be more common at high-volume centers in the context of a multidisciplinary approach. Although we are unable to quantify the rate of planned positive margins using the NCDB, we expect that inclusion of these cases would artificially inflate the margin positive rate at 3T centers and bias our results towards no difference between 1T and 3T centers. In reality, the true negative margin rate may be even higher at 3T centers than reported in this analysis.

Our study has limitations typical of large database studies. Incomplete clinical data as well as lack of granularity can bias our results. The NCDB does not report the cause of death; hence, our endpoint for survival analysis was overall survival and not the oncologically more relevant disease-specific survival. We are also unable to study local recurrence rates and correlate these with margin status or overall survival as recurrence information is not available in the database. Moreover, other measures highly relevant to extra-abdominal STS care such as limb function and treatment-related morbidity are not captured by the NCDB. It is quite possible that 3T centers would have improved local control, limb salvage, limb function, and treatment-related morbidity outcomes compared to 1T centers. The distribution of socioeconomic variables was significantly different between volume terciles. Although these variables were not significant predictors of overall survival, the fact that they were not balanced between groups may have introduced unknown bias. Also, we rely on data abstractors to determine whether surgery was performed for curative intent, and therefore it is not known if R2 resected tumors were for curative or palliative intent. Histologic subtype has been shown to have a significant change in 16% of cases referred to a sarcoma center, suggesting that the histologic subtypes reported by 1T centers may be unreliable in sarcoma cases [22]. To address this issue, we performed a sensitivity analysis by excluding histology from the analyses and found that the results did not significantly change. The hospital population included in the NCDB represents CoC accredited hospitals and does not reflect all inpatient hospitals in the United States. By definition, CoC hospitals have resources that may not be available to other facilities; however, since the NCDB captures 70% of all new cancer diagnoses in the country, it is likely that these results can be generalized. Finally, since our analysis does not include surgeon-specific data, it is unclear if improved outcomes are related more to an individual surgeon's volume rather than a hospital's volume.

So what do we take from this data? First, the inclusion of guideline compliance is important to disentangle the true significance of hospital volume. A prior sarcoma study showed an association between hospital surgical volume and long-term outcomes [11]. When guideline compliance was excluded from our analysis, there was a positive association between hospital surgical volume and long-term outcomes (data not shown). Therefore, it is possible that the inclusion of guideline compliance would have altered the previous study's conclusion. Second, guideline compliance appears to level the playing field between low- and high-volume hospitals with respect to the outcome of survival. Extra-abdominal STS patients who underwent compliant care at a low-volume center achieved an overall survival comparable to those treated at high-volume centers, and those who underwent noncompliant care had statistically worse outcomes. Third, 1T centers treated more stage I tumors and fewer stage III tumors than 3T centers. This suggests that perhaps appropriate triaging to high-volume centers may already be occurring and that high complexity case should be referred to 3T centers.

## 5. Conclusions

High-volume hospitals more often adhere to guidelines. Low-volume hospitals that follow national guidelines appear to achieve comparable survival, but data regarding the association between hospital volume and important outcomes such as local control, limb salvage, limb function, and treatment of related morbidities are lacking. This may help inform the debate on regionalization of sarcoma care. Rather than a focus on volume, centers should encourage expert multidisciplinary consultation and guideline-based treatment for extra-abdominal STS. Currently, adherence to guidelines for all volume terciles remains moderate at best, suggesting a potential avenue to improve outcomes across the board.

## Figures and Tables

**Table 1:** Definition of compliance based on NCCN guidelines for radiation therapy in the treatment of extra-abdominal STS.

	Compliant group	Noncompliant group
Stage I		
T1a-1b and margins R0	No radiation	Yes radiation (overtreatment)
T1a-1b and margins R1 and R2	Yes radiation	No radiation (undertreatment)
T2a-2b	Yes radiation	No radiation (undertreatment)
Stage II and III		
All patients	Yes radiation	No radiation (undertreatment)

**Table 2:** Bivariate associations between volume tercile and patient characteristics.

	1st tercile (*N*=4682)	2nd tercile (*N*=4457)	3rd tercile (*N*=4545)	Total (*N*=13684)	*p* value
Number of hospitals	934	180	44	1158	
Annual number of surgeries	≤3	3.2–< 11	≥11		
Mean annual number of surgeries (range)	2 (1–3)	6 (3–11)	28 (11–81)		
30-day mortality	55 (1.2%)	41 (0.9%)	19 (0.4%)	115 (0.9%)	<0.001
Overall mortality	1676 (35.8%)	1524 (34.2%)	1473 (32.4%)	4673 (34.1%)	0.003
Compliance	1272 (49.8%)	1345 (50.9%)	1582 (53.3%)	4199 (51.5%)	0.03
Age (years)					<0.001
Mean (SD)	58.4 (18.3)	55.2 (17.6)	54.2 (17.7)	56.0 (18.0)	
Median	59	55	55	56	
Sex					0.12
Male	2444 (52.2%)	2335 (52.4%)	2461 (54.1%)	7240 (52.9%)	
Race					<0.001
Missing	44	95	56	249	
White	3040 (85.8%)	3011 (81.4%)	2831 (80.8%)	12057 (83.8%)	
Black	372 (10.5%)	547 (14.8%)	475 (13.6%)	1758 (12.2%)	
Others	131 (3.7%)	141 (3.8%)	199 (5.7%)	570 (4.0%)	
Insurance					<0.001
Missing	99	156	186	441	
No	205 (4.5%)	256 (6.0%)	167 (3.8%)	628 (4.7%)	
Yes	4378 (95.5%)	4045 (94.0%)	4192 (96.2%)	12615 (95.3%)	
Income^†^					<0.001
Missing	215	228	227	670	
<$30,000	570 (12.8%)	640 (15.1%)	506 (11.7%)	1716 (13.2%)	
$30,000–$34,999	768 (17.2%)	730 (17.3%)	776 (18.0%)	2274 (17.5%)	
$35,000–$45,999	1282 (28.7%)	1072 (25.3%)	1241 (28.7%)	3595 (27.6%)	
$46,000+	1847 (41.3%)	1787 (42.3%)	1795 (41.6%)	5429 (41.7%)	
Education					<0.001
Missing	215	228	227	670	
1: 29%+	763 (17.1%)	814 (19.2%)	638 (14.8%)	2215 (17.0%)	
2: 20%–28.9%	978 (21.9%)	955 (22.6%)	943 (21.8%)	2876 (22.1%)	
3: 14%–19.9%	1100 (24.6%)	869 (20.5%)	1026 (23.8%)	2995 (23.0%)	
4: <14%	1626 (36.4%)	1591 (37.6%)	1711 (39.6%)	4928 (37.9%)	
Facility type					<0.001
Community cancer program	919 (19.6%)	22 (0.5%)	0 (0.0%)	941 (6.9%)	
Comprehensive community cancer program	3018 (64.5%)	1730 (38.8%)	575 (12.7%)	5323 (38.9%)	
Academic/research program	744 (15.9%)	2705 (60.7%)	3970 (87.3%)	7419 (54.2%)	
Other specified types of cancer programs	1 (0.0%)	0 (0.0%)	0 (0.0%)	1 (0.0%)	
Charlson/Deyo score					0.004
0	3949 (84.3%)	3762 (84.4%)	3929 (86.4%)	11640 (85.1%)	
1	580 (12.4%)	581 (13.0%)	507 (11.2%)	1668 (12.2%)	
2+	153 (3.3%)	114 (2.6%)	109 (2.4%)	376 (2.7%)	
Primary site					<0.001
Extremity	1964 (41.9%)	2318 (52.0%)	3091 (68.0%)	7373 (53.9%)	
Trunk	2053 (43.8%)	1619 (36.3%)	1083 (23.8%)	4755 (34.7%)	
Head/neck	428 (9.1%)	342 (7.7%)	251 (5.5%)	1021 (7.5%)	
Overlapping	237 (5.1%)	178 (4.0%)	120 (2.6%)	535 (3.9%)	
Histology					<0.001
Liposarcoma	750 (16.0%)	830 (18.6%)	929 (20.4%)	2509 (18.3%)	
Fibrosarcoma	288 (6.2%)	308 (6.9%)	374 (8.2%)	970 (7.1%)	
Leiomyosarcoma	795 (17.0%)	642 (14.4%)	544 (12.0%)	1981 (14.5%)	
MFH	1051 (22.4%)	794 (17.8%)	690 (15.2%)	2535 (18.5%)	
Myxofibroma	26 (0.6%)	27 (0.6%)	35 (0.8%)	88 (0.6%)	
MPNST	156 (3.3%)	175 (3.9%)	221 (4.9%)	552 (4.0%)	
NOS	1616 (34.5%)	1681 (37.7%)	1752 (38.5%)	5049 (36.9%)	
Stage					<0.001
Missing	1757	1558	1357	4672	
Stage I	1571 (53.7%)	1333 (46.0%)	1297 (40.7%)	4201 (46.6%)	
Stage II	716 (24.5%)	687 (23.7%)	764 (24.0%)	2167 (24.0%)	
Stage III	638 (21.8%)	879 (30.3%)	1127 (35.4%)	2644 (29.3%)	
Grade					<0.001
Well differentiated	985 (21.0%)	903 (20.3%)	979 (21.5%)	2867 (21.0%)	
Moderately differentiated	559 (11.9%)	567 (12.7%)	544 (12.0%)	1670 (12.2%)	
Poorly differentiated	986 (21.1%)	1066 (23.9%)	1203 (26.5%)	3255 (23.8%)	
Undifferentiated	539 (11.5%)	629 (14.1%)	974 (21.4%)	2142 (15.7%)	
Cell type not determined	1613 (34.5%)	1292 (29.0%)	845 (18.6%)	3750 (27.4%)	
Tumor size					<0.001
Missing	853	740	613	2206	
<5 cm	1766 (46.1%)	1339 (36.0%)	1317 (33.5%)	4422 (38.5%)	
5–10 cm	1149 (30.0%)	1183 (31.8%)	1250 (31.8%)	3582 (31.2%)	
>10 cm	914 (23.9%)	1195 (32.1%)	1365 (34.7%)	3474 (30.3%)	
Surgical margins					<0.001
Missing	982	743	544	2269	
0: Negative	3071 (83.0%)	3205 (86.3%)	3606 (90.1%)	9882 (86.6%)	
1: Microscopically positive	526 (14.2%)	428 (11.5%)	359 (9.0%)	1313 (11.5%)	
2: Grossly positive	103 (2.8%)	81 (2.2%)	36 (0.9%)	220 (1.9%)	
Surgical margins (amputation cases excluded)					<0.001
Missing	971	735	532	2238	
Negative (R0)	3015 (82.8%)	3078 (85.9%)	3378 (89.6%)	9471 (86.1%)	
Microscopically positive (R1)	523 (14.4%)	424 (11.8%)	357 (9.5%)	1304 (11.9%)	
Grossly positive (R2)	103 (2.8%)	81 (2.3%)	36 (1.0%)	220 (2.0%)	
Chemotherapy					<0.001
Missing	180	119	73	372	
No	4039 (89.7%)	3713 (85.6%)	3589 (80.3%)	11341 (85.2%)	
Yes	463 (10.3%)	625 (14.4%)	883 (19.7%)	1971 (14.8%)	
Radiation					<0.001
Missing	113	85	46	244	
No	3173 (69.4%)	2813 (64.3%)	2612 (58.1%)	8598 (64.0%)	
Yes	1396 (30.6%)	1559 (35.7%)	1887 (41.9%)	4842 (36.0%)	
Extremity STS only	(*N*=1964)	(*N*=2318)	(*N*=3091)	(*N*=7373)	
Amputation rate	70 (3.6%)	139 (6.0%)	242 (7.8%)	451 (6.1%)	<0.001

^†^Median household income for each patient's area of residence as estimated by matching the zip code of the patient recorded at the time of diagnosis against files derived from year 2000 US Census data; ^‡^measure of the number of adults in the patient's zip code who did not graduate from high school as estimated by matching the zip code of the patient recorded at the time of diagnosis against files derived from year 2000 US Census data; MFH, malignant fibrous histiocytoma; MPNST, malignant peripheral nerve sheath tumor; NOS, not otherwise specified.

**Table 3:** Multivariable analysis for overall mortality.

Variable	Comparison	Adjusted HR	95% CI		*p* value
Volume	2nd versus 1st tercile	0.94	0.85	1.04	0.24
	3rd versus 1st tercile	0.92	0.82	1.02	0.12
Age	Per 1-year older	1.03	1.03	1.04	<0.001
Sex	Male versus female	1.20	1.10	1.31	<0.001
Race	White versus black/others	0.92	0.81	1.04	0.18
Insurance	Yes versus no	0.81	0.66	1.01	0.06
Income	$30,000–$34,999 versus < $30,000	0.93	0.79	1.09	0.37
	$35,000–$45,999 versus < $30,000	1.01	0.86	1.19	0.88
	$46,000+ versus < $30,000	0.88	0.74	1.05	0.16
Education	20%–28.9% versus 29%+	0.94	0.81	1.08	0.36
	14%–19.9% versus 29%+	0.89	0.76	1.04	0.13
	<14% versus 29%+	0.88	0.75	1.03	0.11
Comorbidity	1 versus 0	1.14	1.02	1.28	0.02
	2+ versus 0	1.81	1.50	2.18	<0.001
Site	Head/neck versus extremity	1.65	1.38	1.97	<0.001
	Overlapping versus extremity	1.45	1.15	1.83	0.002
	Trunk versus extremity	1.19	1.09	1.31	<0.001
Tumor size	5–10 cm versus < 5 cm	1.51	1.35	1.69	<0.001
	>10 cm versus < 5 cm	2.43	2.16	2.74	<0.001
Grade	Moderately differentiated versus well differentiated	1.70	1.40	2.07	<0.001
	Poorly differentiated versus well differentiated	3.34	2.84	3.94	<0.001
	Undifferentiated versus well differentiated	3.16	2.66	3.76	<0.001
	Cell type not determined versus well differentiated	2.42	2.01	2.91	<0.001
Histology	Leiomyosarcoma versus fibrosarcoma	1.27	1.03	1.57	0.03
	Liposarcoma versus fibrosarcoma	0.84	0.67	1.04	0.11
	MFH versus fibrosarcoma	1.04	0.85	1.28	0.70
	MPNST versus fibrosarcoma	2.15	1.66	2.79	<0.001
	Myxofibroma versus fibrosarcoma	0.83	0.43	1.58	0.56
	NOS versus fibrosarcoma	1.23	1.01	1.51	0.04
Margins	Positive versus negative	1.36	1.23	1.50	<0.001
Chemotherapy	Yes versus no	1.13	1.00	1.27	0.05
Guideline compliant	Yes versus no	0.79	0.73	0.87	<0.001

MFH, malignant fibrous histiocytoma; MPNST, malignant peripheral nerve sheath tumor; NOS, not otherwise specified.

**Table 4:** Multivariable analysis for 30-day mortality.

Variable	Comparison	Adjusted OR	95% CI		*p* value
Volume	2nd versus 1st tercile	0.67	0.28	1.55	0.29
	3rd versus 1st tercile	0.49	0.16	1.31	0.12
Age	Per 1-year older	1.03	1.00	1.06	0.01
Sex	Male versus female	1.28	0.62	2.72	0.45
Race	White versus black/others	1.32	0.46	4.67	0.58
Insurance	Yes versus no	0.71	0.16	6.67	0.66
Income	$30,000–$34,999 versus < $30,000	0.99	0.27	3.60	0.98
	$35,000–$45,999 versus < $30,000	1.51	0.43	5.70	0.47
	$46,000+ versus < $30,000	1.25	0.30	5.50	0.73
Education	20%–28.9% versus 29%+	0.47	0.15	1.39	0.12
	14%–19.9% versus 29%+	0.24	0.06	0.86	0.01
	<14% versus 29%+	0.38	0.11	1.37	0.09
Charlson	1 versus 0	1.06	0.36	2.63	0.90
	2+ versus 0	5.87	2.07	14.86	<0.001
Site	Others (head/neck/overlapping) versus extremity	0.30	0.00	2.46	0.34
	Trunk versus extremity	2.10	0.97	4.81	0.04
Tumor size	5–10 cm versus < 5 cm	1.29	0.37	5.48	0.66
	>10 cm versus < 5 cm	4.06	1.38	15.90	0.007
Grade	Moderately differentiated versus well differentiated	2.39	0.39	12.70	0.24
	Poorly differentiated versus well differentiated	4.78	1.50	19.49	0.006
	Undifferentiated versus well differentiated	3.41	0.97	14.48	0.04
	Cell type not determined versus well differentiated	4.97	1.33	21.85	0.01
Margins	Positive versus negative	3.22	1.54	6.80	<0.001
Chemotherapy	Yes versus no	0.44	0.09	1.47	0.19

**Table 5:** Multivariable analysis for margin negative surgery^∗^.

Variable	Comparison	aOR	95% CI		*p* value
Volume	2nd versus 1st tercile	1.26	1.08	1.47	0.003
	3rd versus 1st tercile	1.77	1.51	2.08	<0.001
Age	Per 1-year older	0.98	0.98	0.99	<0.001
Site	Head/neck versus extremity	0.67	0.50	0.89	0.005
	Overlapping versus extremity	0.68	0.49	0.95	0.03
	Trunk versus extremity	0.92	0.80	1.06	0.25
Tumor size	5–10 cm versus < 5 cm	0.55	0.47	0.66	<0.001
	>10 cm versus < 5 cm	0.37	0.32	0.45	<0.001
Grade	Moderately differentiated versus well differentiated	1.11	0.88	1.40	0.37
	Poorly differentiated versus well differentiated	1.02	0.84	1.23	0.86
	Undifferentiated versus well differentiated	0.98	0.79	1.21	0.85
	Cell type not determined versus well differentiated	1.13	0.90	1.41	0.30
Histology	Leiomyosarcoma versus fibrosarcoma	1.43	1.05	1.94	0.02
	Liposarcoma versus fibrosarcoma	0.89	0.67	1.19	0.45
	MFH versus fibrosarcoma	1.28	0.95	1.72	0.11
	MPNST versus fibrosarcoma	0.64	0.44	0.93	0.02
	Myxofibroma versus fibrosarcoma	0.59	0.27	1.27	0.18
	NOS versus fibrosarcoma	1.07	0.81	1.41	0.65

^∗^Amputation cases excluded (*N*=13,233); MFH, malignant fibrous histiocytoma; MPNST, malignant peripheral nerve sheath tumor; NOS, not otherwise specified.
